# Genomic profile of Toll-like receptor pathways in traumatically brain-injured mice: effect of exogenous progesterone

**DOI:** 10.1186/1742-2094-8-42

**Published:** 2011-05-08

**Authors:** Fang Hua, Jun Wang, Tauheed Ishrat, Wenjing Wei, Fahim Atif, Iqbal Sayeed, Donald G Stein

**Affiliations:** 1 Department of Emergency Medicine, Brain Research Laboratory, Emory University School of Medicine, 1365B Clifton Rd, Atlanta, GA 30322, USA; 2 Department of Medicine, Emory University School of Medicine, Atlanta, GA 30322, USA

**Keywords:** Toll-like receptors, progesterone, traumatic brain injury, inflammation, mouse

## Abstract

**Background:**

Traumatic brain injury (TBI) causes acute inflammatory responses that result in an enduring cascade of secondary neuronal loss and behavioral impairments. It has been reported that progesterone (PROG) can inhibit the increase of some inflammatory cytokines and inflammation-related factors induced by TBI. Toll-like receptors (TLRs) play a critical role in the induction and regulation of immune/inflammatory responses. Therefore, in the present study, we examined the genomic profiles of TLR-mediated pathways in traumatically injured brain and PROG's effects on these genes.

**Methods:**

Bilateral cortical impact injury to the medial frontal cortex was induced in C57BL/6J mice. PROG was injected (i.p., 16 mg/kg body weight) at 1 and 6 h after surgery. Twenty-four hours post-surgery, mice were killed and peri-contusional brain tissue was harvested for genomic detection and protein measurement. RT-PCR arrays were used to measure the mRNA of 84 genes in TLR-mediated pathways. Western blot, ELISA and immunohistochemistry were used to confirm the protein expression of genes of interest.

**Results:**

We found that 2 TLRs (TLR1 and 2), 5 adaptor/interacting proteins (CD14, MD-1, HSPA1a, PGRP and Ticam2) and 13 target genes (Ccl2, Csf3, IL1a, IL1b, IL1r1, IL6, IL-10, TNFa, Tnfrsf1a, Cebpb, Clec4e, Ptgs2 and Cxcl10) were significantly up-regulated after injury. Administration of PROG significantly down-regulated three of the 13 increased target genes after TBI (Ccl-2, IL-1b and Cxcl-10), but did not inhibit the expression of any of the detected TLRs and adaptor/interacting proteins. Rather, PROG up-regulated the expression of one TLR (TLR9), 5 adaptor/interacting proteins, 5 effectors and 10 downstream target genes. We confirmed that Ccl-2, Cxcl-10, TLR2 and TLR9 proteins were expressed in brain tissue, a finding consistent with our observations of mRNA expression.

**Conclusion:**

The results demonstrate that TBI can increase gene expression in TLR-mediated pathways. PROG does not down-regulate the increased TLRs or their adaptor proteins in traumatically injured brain. Reduction of the observed inflammatory cytokines by PROG does not appear to be the result of inhibiting TLRs or their adaptors in the acute stage of TBI.

## Background

Traumatic brain injury (TBI) comprises a cascade of events that begins with a primary neuronal/glial insult and progresses to further proximal and distal cell loss. At the cellular level, the major effectors in this cascade are the activation of inflammatory responses including the release of cytokines, chemokines and adhesion molecules, and the recruitment of leukocytes [1-6].

The neuroprotective action of progesterone (PROG) in TBI has been extensively studied by our laboratory and many others [7-11]. Given after a TBI, PROG has been shown to attenuate cerebral edema, improve spatial learning performance, reduce sensory neglect, and inhibit the increase of some inflammatory cytokines and inflammation-related factors, such as IL-1β, TNF-α, CFC3, GFAP and NFкB [7-14]. How the various immunological and inflammatory responses in traumatically injured brain are activated and regulated, and the mechanisms underlying the neuroprotective effect of PROG against TBI, have not been fully elucidated.

**Figure 1:**
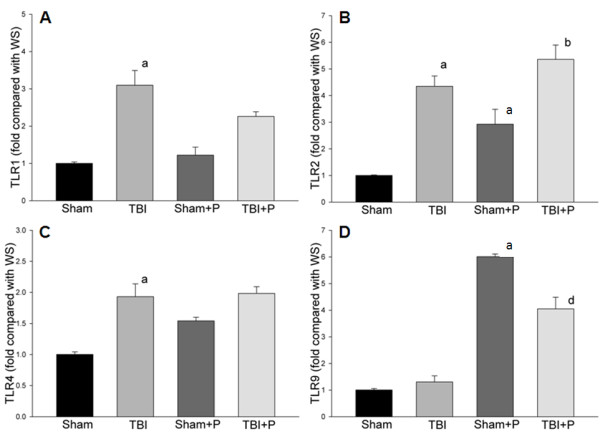
**Fold changes of TLRs after TBI and treatment with PROG**. The figure shows the fold changes of Toll-like receptors (TLRs) after traumatic brain injury (TBI) compared with the sham group. In the 10 detected TLRs, compared to sham controls, TLR1 and TLR2 genes were significantly up-regulated (**A**, **B**). The TLR4 gene was up-regulated 1.9-fold (**C**). Progesterone (PROG) did not inhibit the increased TLRs, but significantly up-regulated the expression of TLR9 in mice with and without TBI (**D**). Note: a: compared with Sham; b: compared with Sham+P; d: compared with TBI; ≥2-fold, p < 0.05; Sham: n = 3; Sham+P: n = 3; TBI: n = 5; TBI+P: n = 3.

The important regulators that mediate much of the inflammatory cascade are the Toll-like receptors (TLRs), a trans-membrane receptor family that has emerged as a key factor in the triggering of antimicrobial host defense responses by the innate immune system (Figure 1). At present, at least 10 human and 12 mouse TLRs have been reported to be expressed in a variety of mammalian immune-related cell types as well as non-immune cells including microglia, astrocytes, oligodendrocytes and neurons [15-21]. Each TLR recognizes its distinct ligands derived from various microorganisms initiating immunological and inflammatory responses [22,23]. For instance, TLR2 recognizes peptidoglycans (PGN), TLR4 recognizes lipopolysaccharide (LPS), and TLR9 recognizes viral CpG DNA [23]. Stressed and damaged cells can also release endogenous ligands which activate TLR-mediated signaling. Thus, immune inflammatory responses can be activated by an injury without the presence of invading pathogens [24]. Upon activation by exogenous and endogenous ligands, TLRs recruit a set of adaptors, including MyD88, TIRAP, TRIF and TRAM, and then activate downstream kinases and cellular signaling pathways which regulate the expression of genes triggering inflammation and immunity [23,25,26].

Involvement of TLR-mediated signaling has been reported in central nervous system (CNS) diseases such as multiple sclerosis [27], Alzheimer's disease, Parkinson's disease, amyotrophic lateral sclerosis [28] and ischemic brain damage [25,29-32]. Recently, increased mRNAs for TLR1, 2, 4, 5, 7 and molecules associated with TLR signaling was reported in spinal cord after injury [33]. However, less is known about the genomic profile of the TLR-mediated pathways in the traumatically injured brain. Such knowledge could contribute to a better understanding of the pathology of secondary damage after brain injury and to the mechanisms of action of PROG in enhancing neuroprotection after injury. In the present study, RT^2 ^Profiler PCR arrays were used to detect the mRNA of 84 genes implicated in regulating TLR pathways in mice with TBI. We then examined the effect of PROG on the expression of these genes. The protein expression of TLR2, TLR9, Ccl-2 and Cxcl-10 was also investigated.

## Methods

### Animals

Male C57BL/6J mice with body weights between 25 and 30 g were obtained from the Jackson Laboratory and maintained in the Division of Laboratory Animal Resources at Emory University. The experiments outlined in this manuscript conform to the Guide for the Care and Use of Laboratory Animals published by the National Institutes of Health (NIH Publication No. 85-23, revised 1996). Animal care and experimental protocols (150-2009) were approved by the Emory University Committee on Animal Care. Mice were assigned to one of four groups: sham-injury control plus vehicle (Sham, n = 11), TBI plus vehicle (TBI, n = 15), sham-injury plus PROG treatment (Sham+P, n = 11), and TBI plus PROG treatment (TBI+P, n = 13). PROG was purchased from Sigma-Aldrich Co. (St. Louis, MO; Cat No. 7556). Based on the previous dose-response studies of PROG in animals [14,34], we used a dose of 16 mg/kg body weight. PROG was injected intraperitoneally (IP) in Sham+P and TBI+P groups at 1 and 6 h after surgery. The sham and TBI-only groups were injected with the same volume of vehicle.

### Induction of TBI

Bilateral cortical impact injury (CCI) to the medial frontal cortex (MFC) has been previously described [35], and was modified for mice in this experiment as follows: mice were weighed and anesthesia was induced by 5.0% isoflurane (Webster Veterinary Supply, Inc., Sterling, MA). The scalp was shaved using electric hair clippers and the exposed skin disinfected with iodine tincture. The trachea was intubated with a 20-gauge intravenous catheter. Anesthesia was maintained by inhalation of 1.5% to 2% isoflurane driven by oxygen flow by a Laboratory Animal Anesthesia System (VetEquip, Pleasanton, CA). The lungs were mechanically ventilated at a rate of 110 breaths per min with a delivered tidal volume of 0.5 ml. The mice were mounted in a stereotaxic device with their heads in a horizontal position. Body core temperature was maintained at 37°C with a homeothermic heating blanket system (TC-1000; CWE Inc., Ardmore, PA). Blood SpO_2 _was monitored and maintained at levels ≥90% using a SurgiVet™ pulse oximeter (model V3304; Waukesha, WI). Under aseptic conditions, the mice were secured in a prone position, and a midline sagittal scalp incision was made using sterile technique. A contusion was produced on the exposed cortex (the anterior-posterior coordinate for the epicenter of the injury was bregma - 1.0 mm) using a magnetic impactor device. The impactor, containing a 2.0 mm diameter rod tip, compressed the cortex at 3.5 m/s to a depth of 1 mm with a dwell time of 100 ms. The incision was sutured and the mice were allowed to achieve spontaneous respiratory effort prior to extubation. Sham control animals underwent surgical procedures identical to those for the TBI group, but no impact was delivered. After awakening, the mice were placed in a holding cage at 31°C for 2 hours and then returned to their housing in the animal care room.

### Preparation of tissue for extraction of RNA

Since most inflammatory factors increase within 24 h after initial insult to brain and PROG is known to inhibit many of these inflammatory factors in the early stage of TBI, the time point of 24 h after injury was selected for tissue analysis. Thus, 24 h after TBI and sham operations, 3 mice from the sham group, 5 mice from the TBI group, 3 mice from the Sham+P group and 3 mice from the TBI+P group were killed and peri-contusional brain tissue (brain area within 3 mm of the epicenter of the injury) was harvested using a biopsy puncture (6 mm diameter), snap-frozen in liquid nitrogen and stored at -80°C for PCR array. To confirm the expressed genes are from brain or blood-derived cells, 3 mice in each group were euthanized, perfused with ice-cold PBS via the ascending aorta, and then peri-contusional brain tissue (brain area within 3 mm of the epicenter of the injury) was harvested.

### Extraction of RNA

RNA was extracted from the tissue using the TRIzol^® ^protocol (15596-026; Invitrogen, Carlsbad, CA). For PCR array, after ethanol precipitation, the RNA was further cleaned using SABiosciences's RT2 qPCR-Grade RNA Isolation Kit (PA-001; Frederick, MD) according to the manufacturer's protocol. RNA quality was analyzed and met the required criteria for RT-PCR arrays and RT-PCR TaqMan^® ^Gene Expression Assays.

### Real-time PCR arrays

RT-PCR arrays were performed by SABiosciences. cDNAs were synthesized using an RT^2 ^First Stand Kit (SABiosciences) according to its protocol. The reaction was performed at 42°C for 15 min and then stopped immediately by heating at 95°C for 5 min. Mouse TLR signaling pathway PCR array kits were purchased from SABiosciences (PAMM-018). This kit profiles the expression of 84 genes related to TLR-mediated signal transduction and 5 housekeeping genes (GUSB, HPRT1, HSP90ab1, GADPH and ACTB). Genes are functionally grouped as TLR, adaptor and interacting proteins, effectors, downstream pathways and target genes, including NFκB pathway, JNK/p38 pathway, NF/IL6 pathway, IRF pathway, and the regulation of adaptive immunity group (Table 1). A negative control for genomic DNA and contaminating RNA was also conducted in each sample. Amplification, data acquisition, and the melting curve were carried out by the ABI 7900 Real Time PCR system (Applied Biosystems, Foster City, CA). The PCR cycling program was set as follows: stage 1: 95°C for 10 min, stage 2: 95°C for 15 sec followed by 60°C for 1 minute repeated for 40 cycles, and stage 3: 95°C for 15 sec, 60°C for 15 sec and 95°C for 15 sec. The cycle threshold (Ct) and melting curve of each gene were automatically established and recorded by the software. The delta Ct (ΔC_t_) method was used for PCR array data analysis. The normalized ΔC_t_) for each gene of interest (GOI) was calculated by deducting the average Ct of the 5 housekeeping genes (HKG) from the Ct of each GOI. Then the double delta Ct (ΔΔCt) for each GOI was calculated by deducting the average ΔCt of GOI in the sham group from the ΔCt of each GOI. The fold-change of each GOI compared to the sham group was calculated as 2^-ΔΔCt^.

**Table 1:** The detected genes of interest (GOI)

		Fold Changes		
		
Genes	RefSeq	TBI/Sham	Sham+P/Sham	TBI+P/TBI
**Toll-like receptors**				
TLR1	NM_030682	2.99*	1.18	-1.33
TLR2	NM_011905	4.28*	2.82*	1.24
TLR3	NM_126166	1.20	1.82	1.20
TLR4	NM_021297	1.89*	1.54	1.05
TLR5	NM_016928	-1.28	1.49	1.47
TLR6	NM_011604	-1.12	1.88	1.70
TLR7	NM_133211	1.52	2.04	1.78
TLR8	NM_133212	1.79	3.44	1.55
TLR9	NM_031178	1.21	6.01*	3.31*
Muc13	NM_010739	1.15	-1.76	-1.39
**Adaptors & interacting proteins**				
Btk	NM_013482	1.56	1.81	1.24
Cd14	NM_009841	7.51*	1.00	-1.84
Hmgb1	NM_010439	-1.07	1.19	1.12
Hras1	NM_008284	-1.78	1.88	2.74*
Hspa1a	NM_010479	1.97*	1.3	-1.37
Hspd1	NM_010477	1.05	-1.11	-1.21
MD-1	NM_010745	2.51*	1.06	1.02
Ly96	NM_016923	1.08	1.01	-1.07
Mapk8ip3	NM_013931	-1.09	-1.04	-1.06
MyD88	NM_010851	1.33	2.86*	2.65*
Peli1	NM_023324	-1.16	1.77	1.82
Pglyrp1	NM_009402	2.35*	1.64	1.34
Ripk2	NM_138952	1.10	1.18	1.04
Ticam1	NM_174989	-1.99	8.98*	16.18*
Ticam2	NM_173394	2.04*	4.87*	3.76*
Tirap	NM_054096	1.24	2.93	2.04*
Tollip	NM_023764	-1.09	1.12	1.11
**Effectors**				
Casp8	NM_009812	-1.03	2.10*	2.04*
Fadd	NM_010175	1.34	6.02*	4.63*
Irak1	NM_008363	-1.09	2.11	1.82
Irak2	NM_172161	-1.16	2.05*	2.58*
Map3k7	NM_172688	-1.31	-1.01	1.08
Nr2c2	NM_011630	-1.34	1.88	2.03*
Ppara	NM_011144	-1.80	2.40*	2.72*
Eif2ak2	NM_011163	1.80	1.16	-1.37
Ube2n	NM_080560	-1.15	1.02	-1.01
Ube2v1	NM_023230	-1.11	1.44	1.45
**Downstream and target genes**				
***NFκB pathway***				
Ccl2	NM_011333	41.89*	1.15	-2.73*
Chuk	NM_007700	1.01	2.21	1.80
Csf2	NM_009969	1.50	1.67	-1.28
Csf3	NM_009971	3.07*	3.26*	2.32*
Hrb1 (agfg1)	NM_010472	-1.18	1.58	1.52
IKKb	NM_010546	-1.50	2.66*	3.10*
Il1a	NM_010554	2.03*	-1.23	-1.29
Il1b	NM_008361	8.69*	-1.17	-3.43*
Il1r1	NM_008362	2.16*	1.18	-1.28
Il2	NM_008366	-1.10	1.26	-1.45
Il6	NM_031168	5.17*	-2.17*	-2.33
Il10	NM_010548	2.06*	1.91	1.47
Il12a	NM_008351	1.20	1.64	1.44
Map3k1	NM_011945	-1.28	2.70	1.78
Nfkb1	NM_008689	1.03	1.26	1.09
Nfkb2	NM_019408	1.14	3.86*	3.69*
Nfkbia	NM_010907	1.22	1.29	1.35
Nfkbib	NM_010908	1.15	-1.22	-1.18
Nfkbil1	NM_010909	-1.22	5.17*	4.83*
Nfrkb	NM_172766	-1.20	1.65	1.47
Rel	NM_009044	1.11	1.12	1.03
Rela	NM_009045	-1.02	2.61*	2.6*
Tnf	NM_013693	19.31*	-1.05	-1.31
Tnfaip3	NM_009397	1.04	2.07*	2.38*
Tnfrsf1a	NM_011609	2.91*	5.1*	3.31*
Tradd	NM_001033161	1.09	-1.02	-1.14
***JNK/p38 pathway***				
Elk1	NM_007922	-1.25	2.43*	2.13*
Fos	NM_010234	1.67	1.99	1.34
Jun	NM_010591	1.9*	1.56	1.1
Map2k3	NM_008928	1.40	1.28	-1.07
Map2k4	NM_009157	-1.26	-1.37	-1.34
Map3k1	NM_011945	-1.28	2.74	1.78
Mapk8	NM_016700	-1.45	1.73	1.94
Mapk9	NM_016961	-1.31	1.15	1.28
***NF/IL6 pathway***				
Cebpb	NM_009883	2.06*	-1.19	-1.69
Clec4e	NM_019948	6.72*	-1.35	-1.43
Il6ra	NM_010559	1.13	1.38	1.14
Ptgs2	NM_011198	2.33*	1.99*	1.5
***IRF pathway***				
Cxcl10	NM_021274	22.99*	-1.03	-4.46*
Ifnb1	NM_010510	1.03	-1.86	-1.66
Ifng	NM_008337	1.30	1.01	1.19
Irf1	NM_008390	1.62	1.80	1.18
Irf3	NM_016849	0.99	3.12	1.90
Tbk1	NM_019786	-1.17	1.28	1.31
***Adaptive Immunity***				
CD80	NM_009855	1.26	1.15	1.27
CD86	NM_019388	1.20	-1.11	1.00
Traf6	NM_009424	1.24	4.57*	3.19*

Note: Positive fold change indicates up-regulation. Negative fold change indicates down-regulation. *p < 0.05

### TaqMan^® ^Gene Expression Assays

RT-PCR TaqMan^® ^Gene Expression Assays were performed using TaqMan real-time PCR primers and probes for mouse TLR2 (Assay ID: Mm00442346_m1; RefSeq: NM_011905.3), TLR9 (Assay ID: Mm00446193_m1; RefSeq: NM_031178.2), Ccl-2 (Assay ID: Mm00441242_m1; RefSeq: NM_011333.3), Cxcl-10 (Assay ID: Mm00445235_m1; RefSeq: NM_021274.1), and Hprt1 (Assay ID: Mm00446968_m1; RefSeq: NM_013556.2) purchased from Applied Biosystems. The synthesis of cDNA was performed using iScript™ Reverse Transcription Supermix for RT-qPCR (Bio-Rad, #170-8841) according to its protocol at 42°C for 15 min and then stopped immediately by heating at 95°C for 5 min. Real-time PCR was performed on CFX96™ real-time PCR system (Bio-Rad) in 96-well format and 25 μl reaction volume per well using iQ Supermix (Bio-Rad, #170-8862). Hprt1 control was used for normalization. Each sample was measured in triplicate in a single RT-PCR run. The cycling program was set as follows: stage 1: 95°C for 10 min, stage 2: 95°C for 15 sec (for denaturation) followed by 60°C (transcription) for 1 minute repeated for 40 cycles, and stage 3: 95°C for 15 sec, 60°C for 15 sec and 95°C for 15 sec. The double delta Ct (ΔΔCt) for each GOI was calculated by deducting the average ΔCt of GOI in the sham group from the ΔCt of each GOI. The fold-change of each GOI compared to the sham group was calculated as 2^-ΔΔCt^.

### Western blot

Twenty-four hours after surgery, 16 mice (3 from the sham group, 5 from the TBI group, 3 from the Sham+P group and 5 from the TBI+P group) were euthanized, perfused with ice-cold PBS via the ascending aorta until the perfusion buffer was clear from the right atrium, and then peri-contusional brain tissue (brain area within 3 mm of the epicenter of the injury) was harvested. Western blot was performed as previously described [31]. Briefly, proteins were extracted, electrophoresed with SDS-polyacrylamide gel, and transferred onto Hybond ECL membranes (Amersham Pharmacia, Piscataway, NJ). The ECL membranes were incubated with primary antibody followed by incubation with peroxidase-conjugated secondary antibodies. Signals were detected with the ECL system (Amersham Pharmacia) and quantified by scanning densitometry and computer-assisted image analysis. The primary antibodies used were anti-TLR2 (Santa Cruz Biotechnology, Inc.) and anti-TLR9 (Abcam, Cambridge, MA). The specification of the antibodies were confirmed in our previous study and the data provided by the manufacture.

### Enzyme-linked immunosorbent assay (ELISA)

Concentration of Ccl-2 and Cxcl-10 were examined by ELISA using Ccl-2 and Cxcl-10 ELISA assay kits (eBioscience Inc., San Diego, CA). For each sample, 10 μg of extracted protein was used for detection. The procedure followed manufacturer instructions. The absorbance was read on a spectrophotometer with a wavelength of 450 nm and a reference wavelength of 650 nm. The concentrations of Ccl-2 and Cxcl-10 were calculated according to the standard curve and presented as pg/μg protein.

### Immunohistochemistry (IHC) staining

Twenty-four hours after surgery, two mice from each group were euthanized and perfused with ice-cold PBS followed by 4% paraformaldehyde in PBS (PH = 7.4) via the ascending aorta. Brains were removed, post-fixed in 4% paraformaldehyde for 24 h, and then stored at 4°C in a solution of 30% sucrose-PBS for two days. The brains were embedded in OCT and coronal sections were cut 10 μm thick. TLR2 and TLR9 were co-stained with specific markers for neurons (NeuN) and astrocytes (GFAP). Immunofluorescent labeling was done by incubating primary antibodies overnight at 4°C in humidified chambers. The next day slides were rinsed 3 times with TBS and incubated with directly conjugated secondary antibodies for 1 h at room temperature. Slides were coverslipped using aqueous mounting medium with DAPI. The co-localization of TLR2 and TLR9 with specific markers for neurons and astrocytes on the slide was observed and analyzed under a confocal microscope (Zeiss LSM 510). The first antibodies used were rabbit anti-TLR2 (Abbiotec, San Diego, CA), rabbit anti-TLR9 (Abcam), rat anti-GFAP (Invitrogen) and mouse Fluor 488 conjugated anti-NeuN (Millipore, Billerica, MA). The secondary antibodies used were Texas red conjugated donkey antibody to rabbit IgG (Abcam) and Cy2 conjugated goat antibody to rat IgG (Gene Tex, Inc, Irvine, CA). The negative control slides went through the same process except being incubated with primary antibodies, which were replaced by antibody dilute solution.

### Statistical analysis

For PCR array and TaqMan^® ^Gene Expression Assays, the numbers of normalized ΔCt for each GOI were used for group comparison by one-way ANOVA with the All Pairwise Multiple Comparison Procedures (Bonferroni t-test). Genes that were up-regulated or down-regulated by at least two-fold with *p-*value less than or equal to 0.05 were considered significant. The results from western blotting and ELISA were presented as mean ± SE. One-way ANOVA with All Pairwise Multiple Comparison Procedures (Bonferroni t-test) was used to compare differences in groups.

## Results

### Changes in genes in TLR-mediated pathways after TBI

**Figure 2:**
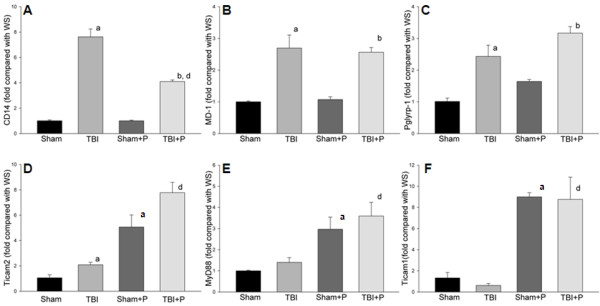
**Fold changes of adaptor and interacting proteins of TLR pathways after TBI and treatment with PROG**. The figure shows the fold changes of adaptor and interacting proteins after TBI compared with the sham group. CD14 (**A**), MD-1 **(B)**, Pglyrp-1 (**C**) and Ticam2 (**D**) were significantly up-regulated). PROG did not significantly inhibit the increased adaptor and interacting proteins, but significantly up-regulated the expression of MyD88 (**E**) and Ticam1 (**F**) in mice with and without TBI compared with sham and TBI groups. Note: a: compared with sham; b: compared with sham + p; d: compared with TBI; ≥2-fold and P < 0.05; Sham: n = 3; Sham+P: n = 3; TBI: n = 5; TBI+P: n = 3.

**Figure 3:**
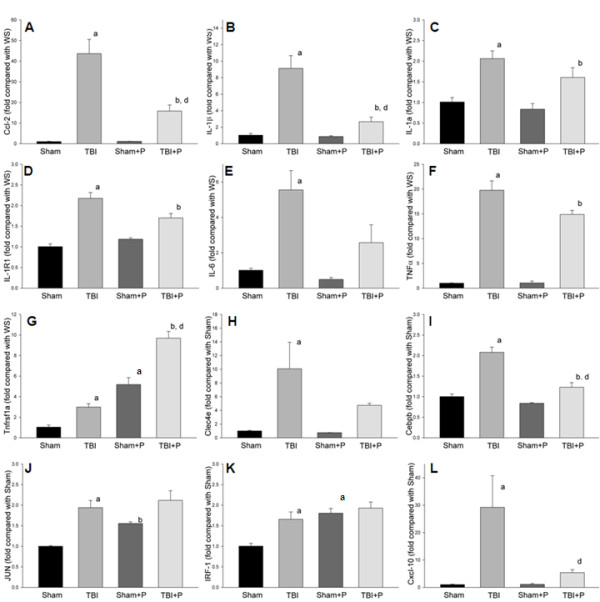
**Fold changes of down-stream target genes in TLR pathways after TBI and treatment with PROG**. The figures show the up-regulated target genes of TLR pathways (Ccl-2, IL-1b, IL-1a, IL1r1, IL-6, TNF, Tnfrs1a, Clec4e, Cebpb, Jun, IRF-1 and Cxcl-10) after TBI compared with shams. Administration of PROG significantly reduced the expression of 3 of the increased genes in brain tissue after TBI (Ccl-2 (**A**), IL-1β (**B**) and Cxcl-10 (**L**) compared to TBI. Note: a: compared with sham; b: compared with sham + p; d: compared with TBI: ≥2-fold, p < 0.05; Sham: n = 3; Sham+P: n = 3; TBI: n = 5; TBI+P: n = 3.

Of the 10 detected TLRs, TLR1 and TLR2 genes were significantly up-regulated in the brain-injured group compared to sham controls (≥2-fold, p < 0.05, Table 1, Figure 1). The TLR4 gene was up-regulated 1.9-fold (p < 0.05). Five of the 17 detected adaptor and TLR-interacting proteins (CD14, Hspa1a, Pglyrp-1, MD-1, and Ticam2) were significantly up-regulated (≥2-fold, p < 0.05, Figure 2). None of the detected effectors of TLR-signaling were up-regulated more than 2-fold compared to the sham-operated group. Thirteen of 40 detected target genes of TLR pathways (Ccl-2, Csf3, IL-10, IL-1a, IL-1b, IL1r1, IL-6, TNF, Tnfrs1a, Cebpb, Clec4e, Ptgs2 and Cxcl-10) were significantly up-regulated (≥2-fold, p < 0.05). Of the 84 detected genes, none was significantly down-regulated more than 2-fold compared to shams (Figure 3).

### Genes down-regulated by PROG in TBI

Administration of PROG significantly reduced the mRNA expression of 3 of the increased genes in brain tissue after TBI (Ccl-2, IL-1b and Cxcl-10; ≥2-fold, p < 0.05, Table 1 and Figure 3). PROG did not significantly down-regulate the increased mRNA of TLRs, adaptor or TLR-interacting proteins, or downstream target genes (Table 1, Figure 1 and 2).

### Genes up-regulated by PROG in brain tissue

Administration of PROG significantly up-regulated 1 TLR (TLR9), 5 adaptor and interacting proteins (Hras1, MyD88, Ticam1, Ticam2 and Tiap), 5 effectors (Casp8, Fadd, Irak2, Nr2c2 and Paea), and 10 downstream target genes (Csf3, Ikbkb, Lta, Nfkb2, Nfkbil1, Rela, Tnfaip3, Tnfrsf1a, Elk1 and Traf6) in TLR pathways compared to the untreated TBI group (Table 1).

### Changes in selected genes and their encoded proteins in TLR-mediated pathways in brain after TBI and PROG treatment

**Figure 4:**
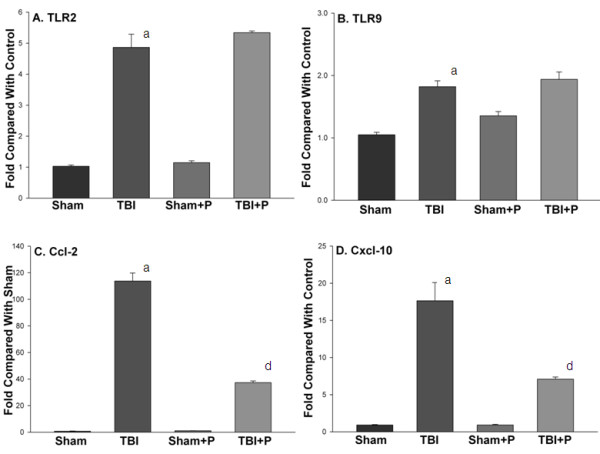
**The mRNA levels of TLR2, TLR9, Ccl-2 and Cxcl-10 in traumatically injured brain with or without progesterone treatment**. The figures show that TLR2 (A), Ccl-2 (C) and Cxcl-10 (D) significantly increased in traumatically injured brain compared with sham controls. Administration of PROG down-regulated the increased mRNA levels of Ccl-2 and Cxcl-10, but did not down-regulate the mRNA levels of TLR2 and TLR9 (B) in sham and injured brain. Note: a: compared with sham; d: compared with TBI: p < 0.05. Sham: n = 3; Sham+P: n = 3; TBI: n = 3; TBI+P: n = 3.

**Figure 5:**
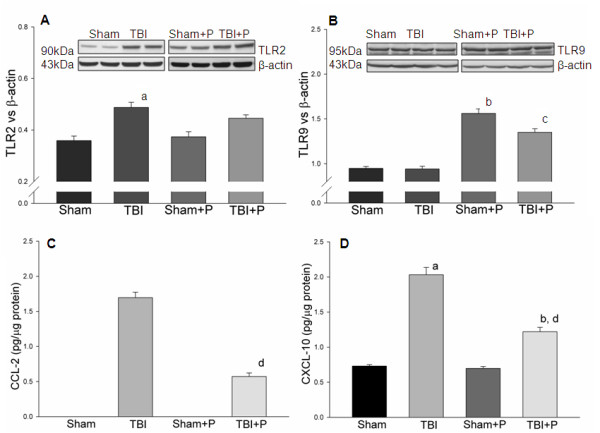
**Expression of TLR2, TLR9, Ccl-2 and Cxcl-10 in traumatically injured brain with or without progesterone treatment**. The figures show that TLR2, Ccl-2 and Cxcl-10, but not TLR9, significantly increased in traumatically injured brain compared with sham controls. Administration of PROG inhibited the increase of Ccl-2 and Cxcl-10, but increased the expression of TLR9 in sham and injured brain. Representative images of western blots are shown on the top of **A **and **B**. Note: a: compared with sham; b: compared with TBI; d: compared with TBI: p < 0.05. Sham: n = 3; Sham+P: n = 3; TBI: n = 5; TBI+P: n = 5.

**Figure 6:**
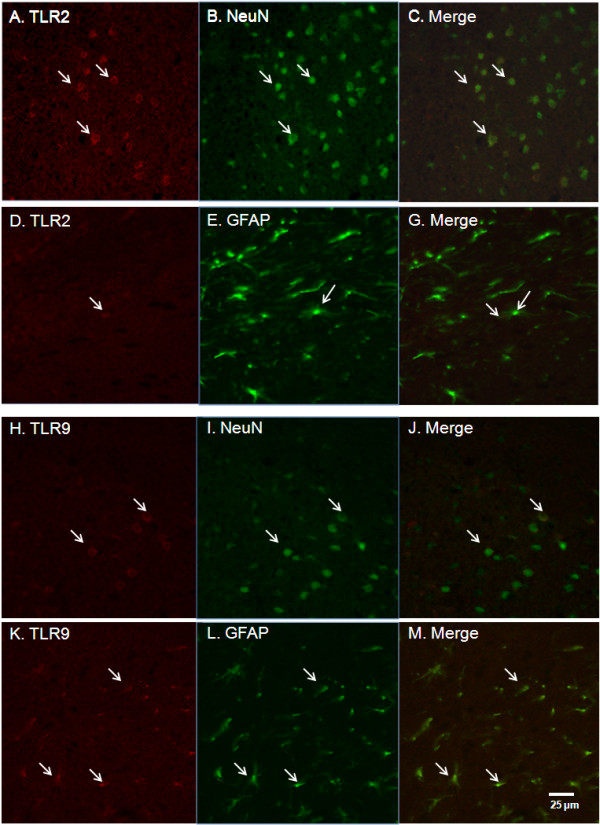
**Immunohistochemical co-staining for TLR2 and TLR9 with NeuN and GFAP**. IHC co-staining of TLR2 and TLR9 with specific markers for neurons (NeuN) and astrocytes (GFAP) was performed and observed under a confocal microscope. The first antibodies used were rabbit anti-TLR2, rabbit anti-TLR9, rat anti-GFAP and mouse Fluor 488 conjugated anti-NeuN. The secondary antibodies used were Texas red conjugated donkey antibody to rabbit IgG and Cy2 conjugated goat antibody to rat IgG. The images show that TLR2 staining co-localized with neurons in the cortex of injured brain (**A, B, C**). However, co-localization of TLR2 with astrocytes was hardly observed in the brain section (**D, E, F**). Weak expression of TLR9 was observed co-localized with neurons in cortex of the injured brain (**G, H, I**). Co-localization of TLR9 with astrocytes was detected in the white matter of periventricular and subcortical regions of the brain (**J, K, L**).

The mRNA expression of selected genes, TLR2, TLR9, Cxcl-10 and Ccl-2, was confirmed using TaqMan^® ^Gene Expression Assays in PBS-perfused brain tissue. The results showed that these genes increased and/or were modified by progesterone in brain tissue after TBI (Figure 4), which are consistent with the results from the PCR-array. To confirm the changes in the proteins encoded by the genes in TLR-mediated pathways, we detected protein levels of TLR2 and TLR9, and the concentrations of Ccl-2 and Cxcl-10 using western blotting and ELISA in brain tissue from mice perfused with PBS. Western blots demonstrated that TBI induced an increase in TLR2, but not TLR9 24 h after injury (Figure 5A, B). Treatment with PROG did not inhibit the increase of TLR2. Rather, PROG increased the expression of TLR9 compared with untreated controls (Figure 5B). Results from ELISA showed that the concentrations of Ccl-2 and Cxcl-10 significantly increased in traumatically injured brain, and that administration of PROG inhibited the increase of Ccl-2 and Cxcl-10 24 hrs after TBI (Figure 5C, D). To determine the cellular location of TLR2 and TLR9 in traumatically injured brain, IHC co-staining was performed using specific anti-bodies for TLR2, TLR9, neuron mark (NeuN) and astrocyte marker (GFAP). TLR2 staining was evident in neurons in the cortex of injured brain (Figure 6A, B, C). However, very little colocalization of TLR2 with astrocytes was observed in the sections (Figure 6D, E, F). Weak expression of TLR9 colocalized with neurons was observed in cortex of the injured brain (Figure 6G, H, I). Colocalization of TLR9 with astrocytes was detected in the white matter of periventricular and subcortical regions of the brain (Figure 6J, K, L).

## Discussion

### TLR-mediated signaling pathways in brain injury

A substantial literature now shows that TLR-mediated signaling pathways play a critical role in the induction and regulation of innate immune/inflammatory responses which contribute to or amplify brain damage following an injury [31,32]. It appears that most inflammatory factors increase within 24 h after initial insult [1-6]. Given soon enough, PROG can inhibit many of these factors [10,36]. Because the injury cascade begins so quickly, we chose to examine TLRs and related genes soon after the initial trauma (24 h).

The present study demonstrated that two TLRs (TLR1 and TLR2) and five adaptor and interacting proteins (CD14, HSPA1a, Pglyrp-1, MD-1, and Ticam2) were significantly up-regulated by TBI (Table 1, Figure 1 and 2). Of the downstream target genes, 12 genes in the MyD88-dependent pathway (Ccl-2, Csf3, Il10, Il1a, Il1b, Il1r1, Il6, TNFa, Tnfrsf1a, Cebpb, Clec4e and Ptgs-2) and only one in the TRIF-IRF pathway (Cxcl-0) increased in brain tissue 24 h after TBI (Table 1 and Figure 3). The results from western blotting and ELISA confirmed that protein levels of TLR2, TLR9, Ccl-2 and Cxcl-10 increased in traumatically injured brain (Figure 5). Immunohistochemistry co-staining demonstrated that TLR2 was expressed mainly in neurons, and that TLR9 was expressed weakly in neurons, but stronger in astrocytes (Figure 6). A similar analysis in traumatically injured spinal cord performed by Kigerl and colleagues [33] showed that mRNA for TLR1, 2, 4, 5, 7, and molecules associated with TLR signaling TLR, such as CD14, MyD88, Traf6, Mal, Tollip, IкBα and NFкB, significantly increased in injured spinal cord. Our results showing that TLR1, TLR2 and CD14 increased after TBI are consistent with Kigerl's report. However, the expression of several genes was different between spinal cord injury and TBI. For example, increased TLR5 TLR7, MyD88, Traf6, IкBα and NFкB were observed in spinal cord, but not in the traumatically injured brain. This variation can be explained by the different anatomic structures and cellular distribution between brain and spinal cord. In addition, the different injury models and time points should also be considered.

Since TLR1 forms heterodimers with TLR2 and then activates down-stream signaling through TLR2, we think that TLR2-mediated signaling is an important pathway in the pathology of TBI. In addition, HSPA1a functions as a molecular chaperone and has been identified as an endogenous ligand of TLR2 or TLR4, which mobilizes NF-kB and induces cytokine synthesis through its interaction with TLR2 or TLR4 [37]. CD14 is an extracellular component of TLR4 which functions as a co-receptor required by the activation of TLR4 induced by lipopolysaccharide (LPS) [23]. MD-1, also known as Ly86, interacting with RP105, plays an important role in the TLR4 signaling pathway [38,39]. Ticam2 physically bridges TLR4 and TICAM-1 and functionally transmits LPS-TLR4 signaling to TICAM-1, which in turn activates IRF-3 [40]. The results show that the 5 adaptor and interacting proteins up-regulated by TBI are closely related to TLR2 and TLR4-mediated signaling. Moreover, the data showing that 12 down-stream target genes in the MyD88-dependent pathway and only one in the TRIF-IRF pathway (Cxcl10) increased in brain tissue, suggest that TBI most likely activates the MyD88-dependent pathway rather than the TRIF-dependent pathway.

We observed that the cytokines, Ccl-2, IL-1b, IL-6, TNF and Cxcl-10 increased more than 5-fold in the TBI group compared to sham controls. The inflammatory cytokines encoded by these five genes are well-known factors that can exacerbate secondary brain injury after TBI. We take our data to indicate that TBI is one of the stresses that up-regulates gene expression in TLR-mediated signaling pathways and that TLR2/TLR4-mediated MyD88-dependent pathways are the most closely implicated in the unfolding pathophysiological processes following TBI.

### PROG affects TLR-mediated signaling pathways after brain injury

Previous studies have indicated that PROG has multiple functions in the CNS, regulating cognition, mood, inflammation, mitochondrial function, neurogenesis and regeneration, myelination, and recovery from TBI (for recent reviews see [9,10,41]). Some PROG-regulated neural responses are mediated directly by PROG receptors (PR), which can be found in every neural cell type in the brain [41]. The PRs regulate gene expression, induce the transduction of signaling cascades, and activate transcription factors [42,43].

Despite the large body of evidence suggesting that PROG protects brain from traumatic injury and improves functional outcomes, a full account of all the mechanisms underlying PROG's plieotropic and neuroprotective effects is far from completed. In the present study, administration of PROG significantly inhibited the up-regulation of some of the genes coding inflammatory cytokines in traumatically injured brain tissue. To our surprise, PROG did not down-regulate any of the detected TLRs, adaptor proteins, effectors, and other target genes in the TLR pathways. We take this finding to mean that PROG can down-regulate some, but not all, of the genes that encode inflammatory cytokine expression after TBI. This effect may be mediated through direct genomic regulation or through a variety of other non-genomic mechanisms [41-44]. Clearly, PROG did not affect the inflammatory response by inhibiting TLRs and their adaptor mRNA. PROG treatment did significantly increase gene expression of one TLR (TLR9), 5 adaptor/interacting proteins, 5 effectors and 10 downstream target genes (Table 1). Interestingly, most of the genes that were increased by PROG were not increased by TBI itself. The results from western blot and ELISA also confirmed that PROG increased the expression of TLR9 but not TLR2, and that PROG inhibited the increase of Ccl-2 and Cxcl-10 induced by TBI (Figure 5). Apparently PROG can regulate a number of TLR pathways by activating different functional gene groups that could have more indirect effects on the injury cascade and neural repair. It has been reported that activation of TLRs, such as TLR9, induced a neuroprotective effect on ischemic brain, which is possibly mediated by suppressing proinflammatory mediators and increasing neuroprotective factors [45]. Up-regulating genes in TLR-pathways may be one of a number of different mechanistic pathways by which PROG can protect the damaged brain from further tissue loss and subsequent functional deterioration. Given the fact that there are different pathways to neuroprotection, there is good reason to consider that combination therapies that would address the different signaling cascades initiated by CNS damage, would be a better approach than hoping to find a single agent that could 'do it all'.

In future research we will need to identify the cellular distribution and specific role of each functional gene regulated by PROG following TBI. It is also important to recognize that TBI and related disorders like stroke have substantial systemic effects that, depending on severity, are not limited to the brain [46,47]. There are now over 180 publications from around the world using more than 20 different injury models in 4 species, including humans, demonstrating the neuroprotective effects of PROG and its metabolites, so the effectiveness of the neurosteroid is clear despite the fact that all of its mechanisms of action are not yet known. Our findings demonstrate that PROG protects brain from injury via multiple mechanisms, such as including inhibition of neurotoxicity factors, mediating TLR-pathways and indirectly activating neuroprotective signaling.

## Conclusion

Our study shows that TBI can increase gene expression in TLR-mediated pathways, and profiles some of this gene expression. Administration of PROG reduced the up-regulation of genes encoding inflammatory cytokines contributing to TBI, and mediated TLR signaling pathways.

## List of Abbreviations Used

Cebpb: CCAAT/enhancer-binding protein beta; CD14: CD14 antigen; Ccl-2: chemokine (C-C motif) ligand 2; Cxcl-10: chemokine (C-X-C motif) ligand 10; Csf3: colony-stimulating factor 3; Clec4e: C-type lectin domain family 4, member e; PrKr: eukaryotic translation initiation factor-2-alpha kinase-2; HSPA1a: heat shock protein 1A; IRF-3: interferon regulatory factor 3; Il: Interleukin; Il1r1: Interleukin 1 receptor 1; LPS: Lipopolysaccharide; MyD88: myeloid differentiation primary response gene 88; MD-1 or Ly86: myeloid differentiation 1; NFкB: nuclear transcription factor kappa B; PGN: peptidoglycans; Pglyrp-1: PGN recognition protein-1; Ptgs-2: Prostaglandin-endoperoxide synthase 2; RIPK2: receptor-interacting serine-threonine kinase-2; TRAF-6: TNF-receptor-associated factor-6; Tnfrsf1a: TNF receptor superfamily member 1A; TBI: traumatic brain injury; Ticam2: TIR domain-containing adapter molecule 2; TRIF or Ticam-1: TIR-domain-containing adapter-inducing interferon-β; TLR: Toll-like receptor; TNFα: tumor necrosis factor α.

## Competing interests

DGS is entitled to royalty from products of BHR Pharma related to research on progesterone, and may receive research funding from BHR, which is developing products related to this research. In addition, the author serves as consultant to BHR and receives compensation for these services. The terms of this arrangement have been reviewed and approved by Emory University which receives the largest share of all licensing fees in accordance with its conflict of interest policies. Other authors declare that they have no conflicts of interest.

## Authors' contributions

FH conceived and designed the study, performed surgery, collected data and wrote the manuscript. JW, TI, FA and IS performed the experiments and reviewed the manuscript. WW participated in the statistical analysis. DGS provided critical advice and worked on the manuscript. All authors read and approved the final manuscript.
